# Cardiac arrhythmia in COVID‐19 patients

**DOI:** 10.1111/anec.13105

**Published:** 2024-02-09

**Authors:** Lei Yu, Ying Liu, Yanjing Feng

**Affiliations:** ^1^ Department of Cardiology Jinan Third People's Hospital Jinan China; ^2^ Department of Cardiology Shandong Second Provincial General Hospital Jinan China

**Keywords:** arrhythmia, heart, infection, SARS‐CoV‐2

## Abstract

The coronavirus disease 2019 (COVID‐19) was first introduced in December 2019, which is known as severe acute respiratory syndrome caused by coronavirus‐2 (SARS‐CoV‐2) that is a serious and life‐threatening disease. Although pneumonia is the most common manifestation of COVID‐19 and was initially introduced as a respiratory infection, in fact, the infection of COVID‐19 is a subset of complications and damage to various organs. There are several reports of cardiac involvement with COVID‐19. A wide range of cardiac complications may occur following COVID‐19 infection, including systolic heart failure, myocarditis, pericarditis, atrial and ventricular arrhythmias, and thromboembolic events. There are various hypotheses about the pathophysiology of cardiovascular involvement by this virus. At the top of these hypotheses is the release of cytokines to the heart. Although there are other assumptions, considering that one of the causes of death in patients with COVID‐19 is arrhythmia. It is necessary to know correctly about its pathophysiology and etiology. Therefore, in this study, we have reviewed the articles of recent years in the field of pathophysiology and etiology of arrhythmia in patients with COVID‐19 infection. The purpose of this study was to provide a basis for a correct and more comprehensive understanding of the pathogenesis of arrhythmia in patients with COVID‐19 infection.

## Graphical abstract

The coronavirus disease 2019 (COVID‐19) is known as SARS‐CoV‐2 infection and is a serious and life‐threatening disease. There are several reports of cardiac involvement with COVID‐19. There are various hypotheses about the pathophysiology of cardiovascular involvement by this virus. At the top of these hypotheses is the release of cytokines to the heart. Considering that one of the causes of death in patients with COVID‐19 is arrhythmia.

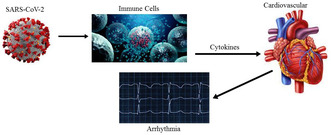

## 1 INTRODUCTION

Severe acute respiratory syndrome caused by the coronavirus‐2 (SARS‐CoV‐2) was first reported in December 2019 (Jee, 2020). At first, it was thought that the SARS‐CoV‐2 infection is a respiratory disease, but later, it was seen that it has a wide range of complications. The most common non‐respiratory complications of the coronavirus are cardiac arrhythmias (Leung et al., 2020; Wang, Hu, et al., 2020; Zhou, Yu, et al., 2020). Arrhythmia increases the mortality rate in patients with the coronavirus disease 2019 (COVID‐19) infection. The occurrence of cardiac arrhythmia has been seen even in patients with COVID‐19 infection who have no history of cardiovascular disease (Liu, Blet, et al., 2020; Ruan et al., 2020). The COVID‐19 virus either directly causes myocardial damage or causes arrhythmia by stimulating the inflammatory response (Babapoor‐Farrokhran et al., 2020; Kochi et al., 2020). In one of the largest available survey, which included 4526 hospitalized patients with COVID‐19, 18.27% of patients developed arrhythmias due to COVID‐19 infection, 22.6% of all arrhythmia cases were bradyarrhythmias, and the rest were tachyarrhythmias. Among the tachyarrhythmias, 81.8% were atrial arrhythmias and 20.7% were ventricular arrhythmias (Coromilas et al., 2021). And these results have been confirmed in smaller studies. In general, the incidence of arrhythmia in COVID‐19 patients has been reported between 10% and 20%. And interestingly, in a retrospective study in China, it was reported that about 7.3% of people mentioned palpitations as the first symptom of contracting the COVID‐19 virus (Liu, Fang, et al., 2020). Zhao et al. (2021) showed that the most common cardiovascular complications caused by the COVID‐19 virus are myocardial damage (21.2%), arrhythmia (15.3%), heart failure (14.4%), and acute coronary syndrome (1%). The probability of death due to arrhythmia was 8.47%, myocarditis 7.61%, and heart failure 3.40%. At the same time, many studies showed that the occurrence of arrhythmia in critical patients is higher than in noncritical patients (Hou et al., 2020). Considering the importance of cardiovascular disease, especially arrhythmia, and its effects in worsening the prognosis of patients with COVID‐19 infection, in this study, we investigate the mechanisms involved in the occurrence of arrhythmia after contracting the COVID‐19 virus so that with a better understanding of its pathophysiology, we can provide better treatments and solutions to control these complications in the future.

## 2 COVID‐19 AND ARRHYTHMIA

Arrhythmias are a potentially life‐threatening complication of COVID‐19 infection. Arrhythmia has been reported in 44% of patients admitted to the intensive care unit (ICU) and 17% of patients admitted to non‐intensive care departments (Long et al., 2020). Induced pro‐inflammatory states, ischemia due to increased demand, myocarditis, and prolonged drugs can cause arrhythmia in COVID‐19 patients. Patients with a history of cardiovascular problems are more prone to arrhythmia. There are many manifestations of arrhythmia in patients with COVID‐19. A series of arrhythmia cases caused by SARS‐CoV‐2 infection include bradyarrhythmias and tachyarrhythmias. An important group of arrhythmias is known as bradyarrhythmia. Bradyarrhythmia can be due to cardiac conduction system defects, transient mechanisms (vagal stimulation, ischemia, and inflammatory activities), and drug side effects (dihydropyridine calcium channel blockers, beta blockers, digoxin, amiodarone, etc.). Bradyarrhythmia can be due to defects in sinus node or ventricular node. Types of sinus node defects included as follows: sinus bradycardia, first‐ and second‐degree sinus block, and sinus arrest or exit block. Types of atrioventricular (AV) node defects included as follows: first‐degree block, second‐degree atrial block (includes Mobitz 1 and Mobitz 2 [Mobitz 1 exhibits the Wenckebach phenomenon, is usually a benign rhythm, causing minimal hemodynamic disturbance and with low risk of progression to third‐degree heart block. Mobitz 2 is characterized by sporadically occurring blocks, without any Wenckebach phenomenon]), and complete heart block. Several studies have reported the incidence of persistent defects in the cardiac conduction system and the eventual need for a pacemaker in COVID‐19 patients (Mehrabinasab & Athari, 2022, 2023). But Studies have shown that the most common bradyarrhythmias in COVID‐19 patients are severe sinus bradycardia and complete heart block (Gopinathannair et al., 2020). Lao et al. (2022) reported that the incidence of new AV block in COVID‐19 patients was 5.5%, most of which were benign.

Tachyarrhythmias are other important types of arrhythmias in COVID‐19 patients, and repeated studies have shown that the most common tachyarrhythmia in these patients is atrial fibrillation (AF) (Gopinathannair et al., 2020). The cause of AF and flutter in COVID‐19 patients is not known properly (Bhatla et al., 2020). Older studies have shown that in critically ill patients with SARS or septicemia, AF is common and has a higher incidence than in the general population. More severe forms of SARS‐CoV‐2 infection can contribute to the creation and continuation of this arrhythmia. Unfortunately, it is more difficult to maintain sinus rhythm in the presence of severe inflammation, electrolyte and metabolic disorders, and increased sympathetic tone. Repeated meta‐analyses have shown that the probability of AF in COVID‐19 patients is about 8%–13%, and this is equal to the probability of AF in influenza patients (12%). This information shows that AF is not specific to COVID‐19 patients and can occur in conditions of systemic inflammation with any cause (Li et al., 2021; Musikantow et al., 2021; Romiti et al., 2021). A retrospective study that examined the data of COVID‐19 patients with arrhythmias showed that 81.8% of those with arrhythmias had atrial arrhythmias, especially AF and atrial flutter and supraventricular tachycardias (VT) (Coromilas et al., 2021).

Ventricular arrhythmia is still the leading cause of death from cardiovascular diseases (Weiss et al., 1991). In a study, it was seen that 6% of patients with COVID‐19 had prolonged QTc (corrected QT; more than 500 ms) (Richardson et al., 2020). These patients were at risk of ventricular arrhythmias. These results indicated that COVID‐19 may be associated with an increased risk of ventricular arrhythmias and sudden death. Also, some studies suggested ventricular arrhythmias as the main cause of death in hospitalized patients with COVID‐19 without the previous history of structural heart disease (Abrams et al., 2020). The most devastating manifestation of ventricular arrhythmias is sudden cardiac death. The risk of VT and ventricular fibrillation is higher in patients with more severe disease, which is reflected by greater severity of metabolic disturbances, hypoxemia, neural and inflammatory stress, and greater myocardial damage. Furthermore, due to infection, this condition may be even more complicated in patients with previous cardiovascular disease or ventricular arrhythmias. In a study, it was seen that the higher the troponin level, the higher the probability of ventricular arrhythmia. In fact, an increase in troponin can be an indicator of the severity of COVID‐19 and is related to hemodynamic instability and cardiac arrhythmia (Bhatla et al., 2020).

### 2.1 Mechanisms of arrhythmias in COVID‐19 patients

Risk factors of arrhythmia in COVID‐19 patients may include hypoxia, which can occur due to pneumonia and airway involvement and pulmonary embolism; imbalance of electrolytes that occurs in the acute phase in COVID‐19 patients; acute myocardial injury due to myocarditis, coronary involvement, acute respiratory distress syndrome (ARDS), and sepsis; and drug interactions. Of course, underlying diseases such as diabetes, high blood pressure, congenital heart problems, heart failure, and thyroid problems increase the incidence of arrhythmia in patients with COVID‐19 (Sciaccaluga et al., 2021). In a study, patients with COVID‐19 underwent heart tissue sampling. It was seen that direct infection of the heart with COVID‐19 occurs and the virus was found in the heart tissue (Lindner et al., 2020). This virus enters the heart cells through angiotensin‐converting enzyme 2 (ACE‐2) receptors and is allowed to multiply in the heart tissue (Zhou, Yang, et al., 2020) Because ACE‐2 receptors are bound by SARS‐CoV‐2, angiotensin II cannot bind to ACE‐2. Therefore, the accumulation of angiotensin II occurs, and finally, angiotensin II acts on ACE type 1 receptors, which leads to a pro‐inflammatory state in the myocardium (Vaduganathan et al., 2020). In the main structure of SARS‐CoV‐2, there are two proteins, namely S1 and S2. S1 plays a major role in binding the virus to ACE‐2. S2 helps the virus to enter the cell. Subunit S1, which is involved in the process of binding to ACE‐2 receptors, determines the recognition of the receptor through its receptor‐binding domain (RBD). By blocking the binding of RBD to ACE‐2 receptors, the virus is unable to enter cells and multiply (Dai & Gao, 2021). Therefore, this receptor can be an excellent therapeutic target.

The main manifestations of direct infection are myocarditis and pericarditis and can cause arrhythmia. Arrhythmia mechanisms in viral myocarditis are numerous, including ischemia caused by endothelial dysfunction, electrolyte imbalance due to the destruction of the membrane of myocardial, and reduced expression of gap proteins in myocardial. The incidence of reentrant arrhythmia in myocarditis can also be associated with angiotensin II receptors. Cardiovascular fibrosis and intensified inflammatory cytokines, especially tumor necrosis factor‐alpha (TNF‐α) and interleukin (IL), also have important appearance in arrhythmia. Changes in the performance of the channels of the cardiac ion and the performance of the fast‐paced potassium channels (IKR) and secondary calcium channels are also effective (Nabeh et al., 2021). During cell membrane damage, IL‐6 increases plakoglobin and the instability of the cell membrane dam and inflammation. The resulting inflammation results in guidance disorders and then arrhythmia (Nabeh et al., 2021). COVID‐19 pericarditis makes the patient susceptible to AF. Also, these patients may be more likely to develop tachycardia‐bradycardia syndrome (Gargano et al., 2021).

### 2.2 Cytokine storm in COVID‐19 infection

In SARS‐CoV‐2 infection, an imbalance between Th1 and Th2 cells results in a cytokine storm, which results in an increase in cytokine release like ILs (1‐b, 2, 6, and 7), interferon‐gamma (IFN‐γ), IFN‐inducible protein‐10 (IP‐10), monocyte chemoattractant protein‐1 (MCP‐1), TNF‐α, and macrophage inflammatory protein‐1A (MIP‐1A) (McGonagle, O'Donnell, et al., 2020). In SARS‐CoV‐2 infection, the imbalance between the cells of th1 and th2 res, resulting in the spread of inflammatory cytokines, causes necrosis and apoptosis, atrial moving. IL‐6 stimulates platelet activation, increases the formation of vascular smooth muscle, and activates endothelial cells (McGonagle, O'Donnell, et al., 2020).

### 2.3 Myocardial ischemia

Excessive inflammatory moods and microvascular dysfunction cause the coronary endothelial system and then ischemia. Intravascular coagulation and immunological activation were discovered in a study of microvascular dysfunction (Dherange et al., 2020). Changes in extracellular potassium levels in ischemic tissues by reducing deployment thresholds can increase electrical conductivity and the occurrence of arrhythmias (Pober & Sessa, 2007).

### 2.4 Malfunction of the endothelium

COVID‐19 decreases the activity of ACE‐2 receptor in cells, stimulates the Kallikrein–Bradykinin pathway, increases the permeability of the blood vessels, and causes endothelial dysfunction (Dherange et al., 2020). Glycocalyx destruction by IL‐1B and TNF‐α activates glucosidases and then reset the type of type 2 hyaluronic acid. Increased hyaluronic acid deposition is associated with arterial hypertension (Pober & Sessa, 2007).

### 2.5 Electrolyte balance disturbances

One of the most important and critical problems in the field of COVID‐19 infection is the occurrence of electrolyte changes. Studies have shown that 5% of hospitalized patients have hypokalemia due to COVID‐19 infection. Hypokalemia can be life‐threatening. The cause of hypokalemia is potassium excretion through urine and digestive tract. In addition, the SARS‐CoV‐2 connection to ACE2 disrupts the functioning of this route, resulting in the disruption of the replacement, especially the potassium (Teuwen et al., 2020).

## 3 ARRHYTHMOGENIC EFFECTS OF CYTOKINES

The severity of the COVID‐19 disease is associated with the effects of cytopathy caused by the virus and the host immune system response (Prompetchara et al., 2020). In patients with COVID‐19, the host immune system can cause a very severe and even fatal inflammatory condition known as cytokine release syndrome (CRS) (Channappanavar & Perlman, 2017). This is a phenomenon of a severe inflammatory response, in which inflammatory cytokines are released quickly and in large quantities in response to infectious stimuli, and the cytokine storm occurs (Wang, Wang, et al., 2020). CRS occurs in the severe shape of COVID‐19 and is one of the most important causes of death. Current studies provide a set of features such as clinical symptoms and laboratory findings to confirm this condition (Caricchio et al., 2021; Webb et al., 2020). The essential mechanisms of the CRS is as follows: virus reproduction in the body stimulates the immune system and causes a highly inflammatory shape of cellular death with lithic planning. In COVID‐19 patients, this process releases anti‐inflammatory cytokines and affects macrophage and lymphocyte's function (McGonagle, Sharif, et al., 2020; Yang, 2020). INF‐1 provides vital help in controlling viral reproduction and promoting adaptive safety systems. Evidence suggests a change in the inherent safety of INF‐1 by COVID‐19. In fact, COVID‐19 affects the host's inherent immunity response and weakens the INF‐1 performance in response to the infection (Huang et al., 2020; McGonagle, Sharif, et al., 2020; Tavakolpour et al., 2020).

Severe inflammation following a chronic virus can cause myocardial damage, acute coronary syndrome, and cardiac arrhythmia. TNF‐α, IL‐6, and IL‐1 affect the performance of potassium and calcium and other ionic channels in cardiomyocytes (Lazzerini, Boutjdir, et al., 2020). The main cause of the inflammatory response in COVID‐19 may be the production of IL‐6. The second hypothesis refers to the comparative safety and the production of antibodies against the virus antigen. In fact, immunoglobulin GS is attached to the S protein and causes the inflammatory waterfall. IL‐8 and MCP‐1 call on monocytes and anti‐inflammatory macrophages in the lung. Evidence suggests that antiviral IgGs are heavily produced as the onset of severe respiratory disease (Zhou, Yang, et al., 2020). The severe forms of COVID‐19 are severely associated with CRS, and CRS is an acute, uncontrolled, and damaging inflammatory response. All body tissues are affected by cytokine storm, including heart tissue (Wang, Hu, et al., 2020). Unfortunately, the level of inflammatory cytokines in COVID‐19 has increased dramatically. G‐CSF, IFN‐γ, IP‐10, TNF‐α, MCP‐1, and MIP‐1α play an important role in COVID‐19 pathogens (Hirano & Murakami, 2020). There is also a strong linear connection between severe lung damage and cytokine levels, including IFN‐γ, IFN‐α2, IL‐1RA, IL‐2, IL‐4, IL‐7, IL‐10, IL‐12, and IL‐17 (Liu, Zhang, et al., 2020). Cytokines play a key role in regulating immunological and inflammatory profiles. In the meantime, IL‐6 is known as the main cytokine in the pathogenesis and the severity of COVID‐19. Therefore, the continuous measurement of IL‐6 levels in people with COVID‐19 is recommended. All immune cells and other cells such as endothelial cells, keratinocytes, fibroblasts, and tumor cells can produce IL‐6. In fact, IL‐6 is a glycoprotein that can act as both pro‐inflammatory and anti‐inflammatory cytokines. IL‐6 plays an important role in B‐cell differentiation and antibody production. Other immunomodulatory roles of IL‐6 are related to the generation of autoreactive CD4 pro‐inflammatory T‐cell responses, stimulation of cytotoxic T‐lymphocyte activity, regulation of T‐helper 17, and regulatory T‐cell balance (Jones et al., 2018; Kimura & Kishimoto, 2010). IL‐6 plays an important regulatory role in homeostasis, such as the acute phase response and hematopoiesis (Huang et al., 2020). High levels of IL‐6 are associated with CRS (Rostamian et al., 2020). In other words, the pathophysiological characteristic of COVID‐19 is closely related to severe inflammatory responses. Therefore, the detection of IL‐6 serum level may predict the progression of the disease of COVID‐19. IL‐1 family members also play a vital role in the inflammatory process. IL‐1α and IL‐1β have pro‐inflammatory effects (Dinarello, 2018). IL‐1 is one of the main elements in the pathogenesis of COVID‐19. IL‐1α is released from endothelial and epithelial cells, but IL‐1β is released by immune cells, including neutrophils, macrophages, and infiltrating monocytes. IL‐1 receptor antagonist (IL‐1 Ra) modulates and prevents excessive IL‐1‐mediated inflammatory responses by binding to IL‐1 receptors (Kim et al., 2013; Zheng et al., 2013).

Inflammatory cytokines, especially TNF‐α, IL‐1, and IL‐6, have important arrhythmogenic effects. These effects are applied through several mechanisms, which are divided into two categories: direct effects and systemic indirect effects. Direct effects include cardiac regeneration. Remodeling is characterized by electrical and structural changes. Electrical changes occur immediately within hours to days after the exposure to cytokines. The cause of these electrical changes is the presence of complex modulating activities of inflammatory cytokines on the expression and function of specific ion channels and gap junctions. These effects cause the occurrence of cardiac inflammatory channelopathies and changes in intracellular proteins. For example, ryanodine receptors are responsible for the transfer of calcium in cardiomyocytes, and cytokines by affecting ryanodine disrupt its activity, and therefore, the balance of electrolytes inside and outside cardiomyocytes is disturbed, and the ground for arrhythmias is created (Lazzerini et al., 2017). Cytokines can also induce structural remodeling that is induced by activating the synthesis of the extracellular matrix responsible for cardiac fibrosis, which is directed by myofibroblasts. Structural remodeling occurs gradually over weeks or months. In general, these phenomena result in prolongation of the action potential duration or QTc interval, which increases aberrant firing, and slow or heterogeneous propagation of electrical impulses throughout the working and conducting myocardium. In turn, this causes tachyarrhythmias and triggered and reentrant bradyarrhythmias, as well as conduction disturbances (Lazzerini et al., 2017). Inflammatory cytokines mediate arrhythmias in COVID‐19 patients. In fact, arrhythmia is not caused by direct heart tissue damage by SARS‐CoV‐2 infection, but the main cause of the event is the systemic inflammatory response to severe SARS‐CoV‐2 infection (Guan et al., 2021; Lazzerini et al., 2022; Musikantow et al., 2021). Postmortem examination shows severe infiltration of interstitial monocytes in the myocardial tissue. In fact, arrhythmia occurs in the context of cytokine storm and excessive inflammatory reactions (Liu, Yang, et al., 2020; Xu et al., 2020). The entry of the virus into the cell and its multiplication causes cytotoxicity, which provokes an inflammatory response in the body (Atri et al., 2020; Chen et al., 2020; Guo et al., 2020). Inflammatory cytokines such as IL‐6 and IL‐8 are released in response to lung injury. With the exacerbation of pneumonia, other inflammatory cytokines such as IL‐10 are also released. In fact, the inflammatory response is related to the severity of the infection, and the possibility of arrhythmia is also related to the severity of the inflammatory response (Zheng et al., 2013). If the virus is not effectively cleared, an excessive inflammatory response occurs. At this time, the cytokine storm causes irreversible damage to various organs, including the heart. IL‐6 can block hERG channels in ventricular myocytes. Also, inflammatory cytokines, by affecting the hypothalamus, over‐activate the sympathetic nervous system, and this makes the person susceptible to arrhythmia (Aromolaran et al., 2018; Lazzerini et al., 2017; Tracey, 2002). A large cohort study showed an independent association between SARS‐CoV‐2 infection and QTc prolongation, and IL‐6 levels in hospitalized patients. This study found that increased IL‐6 levels were directly related to the extent of QTc prolongation (Tracey, 2002). IL‐6 increases QTc by inhibiting CYP3A4 (Lazzerini, Acampa, et al., 2015; Lazzerini et al., 2021). IL‐6 via the inflammation pathway has a strong effect in all organs, and other studies have similar findings and have shown an association between increased IL‐6 with an increased risk of arrhythmia in COVID‐19 patients (Hami et al., 2009; Jirak et al., 2021; Qian et al., 2022; Wang et al., 2021). TNF‐α, IL‐6, and IL‐1 regulate ion channel function and ventricular action potential timing through interactions between potassium and calcium channels (Haddadzadeh et al., 2009; Lazzerini, Acampa, et al., 2020; Lazzerini et al., 2019; Lazzerini, Capecchi, et al., 2015; Masoume Athari et al., 2018; Mehrabi Nasab et al., 2020; Mehrabinasab et al., 2023; Zhiwei et al., 2022). In addition, increased cytokines (especially IL‐6) can reversibly decrease the expression of connexin 43 in the atria and atrioventricular node (Atri et al., 2020; Hirano & Murakami, 2020; Lazzerini et al., 2019; Wang, 2020), thereby causing cardiac electrical remodeling and structural remodeling with increased atrial fibrosis and, as a result, causes AF and AV block.

## 4 CONCLUSION

COVID‐19 is a life‐threatening disease. Strong stimulation of the immune system and the occurrence of cytokine storm cause serious complications in different organs of the body. Extrapulmonary manifestations such as thrombotic complications, myocardial dysfunction and arrhythmias, and acute kidney injury are also commonly found in patients with COVID‐19 infection. One of the most important organs involved is the heart. Cardiac involvement is sometimes accompanied by serious and even life‐threatening complications. Arrhythmia is one of the most important causes of death in extrapulmonary manifestations such as thrombotic complications, myocardial dysfunction and arrhythmia, and acute kidney injury and is also usually found in patients with this virus and patients with severe forms of COVID‐19 infection. Therefore, it is necessary to know the pathophysiology of arrhythmia in patients with COVID‐19 infection. By knowing its pathophysiology, a more correct treatment process can be taken. Cytokine storm and intense stimulation of the immune system by the infection of COVID‐19 by disrupting the function of ion channels in the heart and creating electrical and structural remodeling cause arrhythmia. Therefore, knowing the various aspects of the correlation between immune system disorders and heart problems is the way to design more correct treatment.

## AUTHOR CONTRIBUTIONS

LY, YL, and YF participated in the study and writing of the manuscript.

## CONFLICT OF INTEREST STATEMENT

There is no conflict of interest.

## ETHICS STATEMENT

Not applicable.

## Data Availability

The data that support the findings of this study are available from the corresponding author upon reasonable request.
